# Use and Benefit of Sacubitril/Valsartan in Elderly Patients with Heart Failure with Reduced Ejection Fraction

**DOI:** 10.3390/jcm13164772

**Published:** 2024-08-14

**Authors:** Luis Nieto Roca, Marcelino Cortés García, Jorge Balaguer Germán, Antonio José Bollas Becerra, José María Romero Otero, José Antonio Esteban Chapel, Carlos Rodríguez López, Ana María Pello Lázaro, Mikel Taibo Urquía, José Tuñón

**Affiliations:** 1 Cardiology Department, Son Espases University Hospital, 07120 Balearic Islands, Spain; luis.nietor@quironsalud.es; 2 Cardiology Department, Fundación Jiménez Díaz University Hospital, 28040 Madrid, Spainjtunon@quironsalud.es (J.T.)

**Keywords:** HFrEF, elderly, ARNI, propensity score

## Abstract

**Background:** Heart failure (HF) is a highly prevalent syndrome in elderly subjects. Currently, multiple drugs have shown clinical benefits in patients with HF and reduced ejection fraction (HFrEF). However, evidence is scarce in elderly patients (beyond 75 years old), even more so for the latest drugs, such as angiotensin receptor-neprilysin inhibitors (ARNIs). This study aims to evaluate the use and benefits of ARNIs in elderly patients with HFrEF. **Methods**: A prospective observational cohort study was designed. Patients with left ventricular systolic dysfunction (defined by left ventricular ejection fraction [LVEF] < 40%) and age ≥ 75 years from January 2016 to December 2020 were prospectively included. Patients with an indication for ARNIs at inclusion or throughout follow-up were selected. Clinical, electrocardiographic and echocardiographic variables were collected. **Results**: A total of 616 patients were included, 34.4% of them female, with a mean age of 83.3 years, mean LVEF of 28.5% and ischemic etiology in 53.9% of patients. Only 14.3% of patients were taking ARNIs. After a mean follow-up of 34 months, 50.2% of patients died, and 62.2% had a cardiac event (total mortality or hospital admission due to HF). Multivariate Cox regression analysis showed that the use of ARNIs was independently and significantly associated with lower rates of mortality [HR 0.36 (95% CI 0.21–0.61)], with similar results in relation to all-cause mortality in a propensity-score-matched analysis [HR 0.33 (95% CI 0.19–0.57)]. **Conclusions**: We observed an important underuse of ARNIs in a cohort of elderly HFrEF patients, in which treatment with ARNIs was associated with a significant reduction in mortality. Greater implementation of clinical practice guidelines in this group of patients could improve their prognosis.

## 1. Introduction

Heart failure (HF) is a highly prevalent syndrome among the elderly. The incidence of HF progressively increases with age, reaching around 20% among people over 75 years old [1]. Nowadays, many pharmacological and non-pharmacological therapies have been shown to reduce all-cause mortality and hospitalizations [2]. Clinical trials, however, usually exclude elderly patients or under-represent them, raising concerns about the external validity of their results [3]. Angiotensin-converting enzyme inhibitors (ACEIs), angiotensin receptor blockers (ARBs), beta-blockers (BBs) and mineralocorticoid receptor antagonists (MRAs) are drugs with a longer run in the field of heart failure, which have demonstrated their role in reducing morbimortality and improving left ventricular ejection fraction (LVEF) in elder populations [4,5,6,7,8,9]. Additionally, in the last few years, angiotensin receptor-neprilysin inhibitors (ARNIs) and sodium-glucose transport protein 2 inhibitors (SGLT2i) have become an important therapeutic group for heart failure with reduced ejection fraction (HFrEF). Although we know that neprilysin inhibition in patients with reduced LVEF through ARNIs is associated with significantly improved survival, hospitalizations and quality of life results [10], the evidence in the elderly subpopulation is very scarce. There are no clinical trials focused on ARNIs in this subgroup of patients. We only have sub-analyses of the main ARNI trials (which show clinical benefit in elderly patients) [10,11,12]. On the other hand, even though small observational studies reflect more disparate results, most of them seem to favor the use of ARNIs in terms of safety and clinical efficacy [13,14,15,16]. Nonetheless, the number of publications relating to this concrete subject is relatively small; many of them are subgroup analyses of greater populations, with age intervals far under the clinical practice ones and with significantly smaller populations.

Both ARNIs and SGLT2i appear to suffer from this paucity of evidence. However, there are some observational studies for SGLT2i in elderly patients with HFrEF that look at clinically relevant endpoints like mortality and hospitalization [17], unlike with ARNIs. Thus, the aim of our study is to evaluate the role of ARNI therapy in a population of very elderly patients with HFrEF.

## 2. Materials and Methods

### 2.1. Patient Selection and Study Design

We carried out a single-center, prospective, observational cohort study. We consecutively enlisted 891 patients 75 years of age or older with an LVEF lower than or equal to 40% as measured by 2-dimensional echocardiography from January 2016 to December 2020. A specific database compiled in the cardiac imaging department of our center was used to screen for patients meeting the criteria. We selected patients with a clinical indication for the use of ARNIs at the time of inclusion or throughout follow-up. Ultimately, 616 patients were included in our study. All patients received regular medical supervision according to their symptoms and the indications of their physicians (cardiologists or general practitioners) to optimize treatment. The study design and protocol have been revised and approved by the clinical research ethics committee of our institution (Ref. EO093-18 FJD). This investigation was carried out in accordance with the principles outlined in the Declaration of Helsinki.

### 2.2. Data Collection

All data, including clinical events and death during follow-up, were collected from patients’ electronic health records or, if not available, from telephone interviews with patients or relatives. At the beginning of follow-up, variables recorded included baseline clinical characteristics, cardiovascular risk factors, comorbidities, the glomerular filtration rate (GFR) calculated by the Chronic Kidney Disease Epidemiology Collaboration (CKD-EPI) equation, electrocardiographic (rhythm, heart rate and QRS complex width) and echocardiographic findings, New York Heart Association (NYHA) functional class and the type and dose of cardiovascular drugs. At the end of follow-up, data regarding HF treatment, LVEF and NYHA functional class were gathered.

### 2.3. Outcomes and Follow-Up

The outcomes analyzed in our study were the rate of all-cause mortality and major cardiovascular events. Major cardiovascular events were defined as either death from any cause or admission due to heart failure (HF). HF admission was defined as admission to a healthcare facility lasting > 24 h due to the worsening of HF symptoms and followed by specific treatment for HF (regardless of the cause of decompensation).

### 2.4. Statistical Analysis

Data were subjected to descriptive statistical analysis via frequency measurements (absolute frequencies and percentages) for qualitative variables and using mean and standard deviation for quantitative variables. The magnitude of the effects of the variables was expressed in the form of hazard ratios (HRs) and 95% confidence intervals (CIs). Univariate analysis of the quantitative variables was performed using Student’s *t*-test when the variables were normally distributed and the Mann–Whitney U-test when the distribution was not normal. Qualitative variables were analyzed using χ2 or Fisher’s exact test. Because observational studies do not allow for randomization, we planned two different approaches to avoid potential confounding factors: multivariate Cox proportional hazard and propensity score (PS)-matched analysis. These two analyses were used to determine significant predictors of cardiovascular events and mortality.

First, we performed a multivariate analysis with Cox (backward stepwise) regression. Of all of the baseline variables collected, we selected those with the potential to act as confounding factors. The selection criteria were as follows: first, clinical and biological plausibility and, second, the statistical criterion of Mickey, excluding all of those variables that returned a *p* value > 0.20 on univariate analysis. Multivariate Cox regression analyses were performed for all-cause mortality and cardiovascular events, including clinical variables [age, frailty, NYHA class, previous HF admissions and comorbidities, such as diabetes mellitus, hypertension, chronic lung disease (asthma, chronic obstructive pulmonary disease or sleep apnea/hypopnea syndrome) and peripheral vascular disease (demonstrated atherosclerotic disease in all arteries other than coronary arteries and aorta)], GFR, electrocardiographic variables (presence of sinus rhythm and of a wide QRS complex), LVEF at baseline and follow-up and variables related to therapy [use of ARNIs, ACEI/ARB, BB, MRA, SGLT2i, diuretics and cardiac resynchronization therapy (CRT) or implantable cardioverter-defibrillator (ICD)].

Second, we performed a PS-matched analysis. The PS was calculated with an ordered logistic regression model, taking the ARNI group as the dependent variable and adopting a parsimonious approach. In the first step, all of the following variables were included in the univariate analysis: age, gender, diabetes mellitus, hypertension, GFR, chronic lung disease, peripheral vascular disease, cerebrovascular disease, any degree of cognitive impairment, any degree of functional disability, the ischemic origin of reduced ejection fraction [defined as evidence of significant disease of a major coronary vessel (at least 70% stenosis or, in the case of the left main coronary artery, 50% stenosis) as evidenced by coronary angiography or coronary CT scan, regardless of whether the significant lesion has been revascularized or not], previous HF admission, LVEF and NYHA class I or II (vs. III, IV or not available) at the onset of follow-up. All variables with a *p* value < 0.2 were entered into a multivariate binary logistic regression model, which served to estimate the PS of every patient. Patient matching was performed at a 2:1 ratio with the nearest neighbor method (caliper = 0.2 × standard deviation (SD) [logitPs]). Results are expressed as HR and 95% CIs. Statistical analyses were performed with SPSS version 22.0 (SPSS, Inc, Chicago, IL, USA).

## 3. Results

### 3.1. Baseline Characteristics

During the study period, 616 consecutive patients with an LVEF ≤ 40% were assessed for eligibility. Table 1 shows the baseline characteristics of our population (total population and propensity score-selected population). In terms of sex, 65.6% were male, and the mean age was 83.3 ± 5.1 years. According to cardiovascular risk factors, 81.2% were hypertensive, 35.4% were diabetic, and 57.1% were dyslipidemic. Regarding comorbidities, 53.6% had been diagnosed with chronic kidney disease (GFR < 60 mL/m/m^2^) and 17.7% with chronic lung disease, with chronic obstructive pulmonary disease being the most frequent entity. Ischemic etiology was found in 53.9% of the cases. The mean LVEF was 28.5 ± 7.8%. Further, 86.8% presented with NYHA class I-II, and 58.7% of the subjects were at sinus rhythm at inclusion.

At the end of follow-up, the percentage of patients taking ARNIs was 14.3% of the total cohort. BBs were used by 76.9% of patients, ACEIs/ARBs by 55.5%, MRAs by 40.4% and SGLT2 inhibitors (SGLT2i) by 4.2%. Ivabradine was received by 4.4% of the cohort, and 71.8% of patients underwent diuretic treatment.

We also analyzed the reasons for not using ARNIs in those patients who did not receive this drug by means of a thorough examination of the possible causes throughout the available medical records. Among those patients not receiving ARNIs, 85.8% of the patients did not have a clear contraindication for ARNIs. Within the remaining 14.2%, the main cause for not being treated with ARNIs was impaired renal function (7.2%), followed by drug-induced hypotension (4.7%) and hyperkalemia (0.8%).

### 3.2. Outcomes

After a follow-up of 34 ± 21 months, 309 patients (50.2%) had died, and 383 patients (62.2%) had developed a major cardiovascular event (death or hospitalization for HF). Of the patients who died, the cause of death was cardiovascular in 72 cases (23.3%), and non-cardiovascular causes accounted for 188 deaths (60.8%). We were unable to determine the cause of death in 49 patients (15.8%).

We performed a multivariate Cox regression analysis of our study population to identify significant predictors of total mortality, following the methodology described above. Table 2 shows the results of univariate and multivariate analyses of overall mortality. A multivariate Cox regression analysis revealed that the use of ARNIs was independently associated with lower rates of mortality (HR 0.36 [95% CI 0.21–0.61]) as compared with those who were not receiving sacubitril/valsartan. Similarly, we found a clear relationship between BB intake and reduction in mortality [multivariate Cox regression analysis HR 0.62 (0.48–0.80)]. SGLT2i showed a significant association between its use and reduction in mortality but only for the univariate analysis [HR 0.12 (0.03–0.5)]. On the other hand, neither ACEIs/ARBs nor MRAs were associated with a significant improvement in mortality.

A similar analysis was performed to evaluate potential predictors for total cardiovascular events. ARNI was independently associated with a significant reduction in cardiovascular events [HR 0.69 (0.49–0.99)]. The use of BBs and ACEIs/ARBs was also associated with a significant reduction in events (Table 3).

Finally, a statistical analysis was performed through PS matching, specifically aimed at analyzing the effect of ARNIs in our population. Table 1 shows the baseline characteristics of the selected groups according to the aforementioned methodology with and without ARNI. No significant differences were described between the two groups with respect to age, sex, comorbidities or HF treatments except for the use of SGLT2i (greater in the ARNI group). The PS-matching analysis showed again that the use of ARNIs was associated with a significant reduction in mortality [HR 0.33 (95% CI 0.19–0.57)].

**Figure 1:**
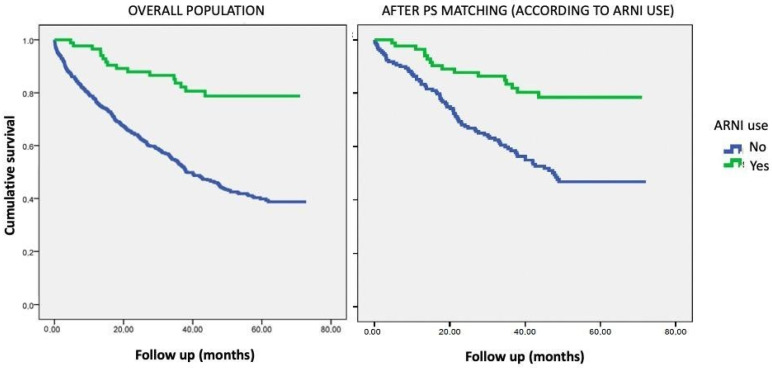
Kaplan–Meier curves for mortality, first showing results for the overall population and then after PS (propensity score) matching according to ARNI use. ARNIs: angiotensin receptor-neprilysin inhibitors.

Kaplan–Meier curves for mortality in the overall population and in the PS-matching population comparing those under treatment with ARNIs to those not treated with ARNIs are shown in Figure 1.

## 4. Discussion

Our study highlights that the use of ARNIs in a cohort of elderly patients (>75 years old) with HFrEF (LVEF ≤ 40%) was associated with increased survival and an improvement in long-term prognosis despite a significant underuse. These results are consistent with those shown in the main trials focused on the general HFrEF population. Nevertheless, our results represent a novel contribution to the body of evidence of ARNI treatment in that it directly evaluates mortality and cardiovascular events in a very elderly cohort instead of relegating them to subgroup or post hoc analyses.

PARADIGM-HF was a guideline-changing trial. It introduced sacubitril/valsartan to the heart failure drug repertoire after showing significant and safe improvements in LVEF, NT-proBNP levels and morbimortality in patients with HFrEF [10]. Additional benefits of ARNIs included an improvement in symptoms, an improvement in quality of life, a reduction in the incidence of diabetes requiring insulin treatment [18], a reduction in the decline of GFR [19] and a reduced rate of hyperkalemia [20]. In this trial, almost 50% were over 65 years old; nevertheless, the proportion of patients > 75 years was just 18.6% (7% > 80 years and 1.44% > 85 years).

Of the overall population included in the PIONEER-HF trial (which demonstrated the efficacy and safety of sacubitril/valsartan in acute decompensated HF), only 36.5% were >65 years old [11]. The median age in the TRANSITION study (showing the feasibility of early initiation of sacubitril/valsartan after acute decompensated HF) was 68 years old, with no specific information regarding the proportion of patients older than 75 years [12].

Most patients in these pivotal trials belong to these “younger elders” between 65 and 75 years of age. The “real” elders are clearly underrepresented (a mere 18.6% versus almost 30% between 65 and 75 in PARADIGM-HF). In addition, current evidence regarding the use of ARNIs in patients > 75 years old and with HFrEF is mostly supported by post hoc or subgroup analysis. In one of them, where patients of the PARADIGM-HF trial were analyzed according to age, a persistent benefit was described beyond 65 years old. However, this was mainly driven by those between 65 and 74 years old, and, even though the results in terms of mortality were relatively stable across the age range, they did not reach statistical significance in the > 75-year-old group. Sacubitril/valsartan was demonstrated to be safe with no greater rate of adverse events; moreover, there was a possible clinical benefit in these patients despite lower doses [21]. On the other hand, the few trials that have directly focused on elderly patients either consider surrogate clinical endpoints or have a very short follow-up period [22].

Considering the lack of elderly-focused trials and the aforementioned underrepresentation in the main HF trials [23,24], real-world registries have shed some more light on the use of ARNIs in the “real elders”. In a real-world series including 205 patients beyond 70 years of age, sacubitril/valsartan was, first, safe, with no significant rate of adverse events compared to the younger group; in addition, its withdrawal was linked with poorer prognosis [25]. A more recent trial based on the FDA adverse events reporting system database showed similar rates of adverse events in patients over 75 years of age when compared with younger peers. [26]. In addition, in a recent large cohort study comparing elderly patients with HFrEF treated with ARNIs versus those receiving ACEIs/ARBs in the UK, the use of ARNIs was associated with a lower risk of hospitalization for HF and all-cause mortality (mainly driven by those patients who previously received a renin-angiotensin system blocker and switched to ARNIs) [27]. Clinical assumptions can also be made regarding our current knowledge of natriuretic peptides and the effects of this inhibition. Along this line, it is well known that they have similar effects in old patients when compared with younger populations [28,29]. Hence, we could expect similar hemodynamic and clinical benefits in this subgroup of patients. Therefore, even though all available data seem to support the use of ARNIs in patients with HFrEF and >75 years old, there is still no solid evidence of their clinical benefit in this population.

On the other hand, HF is a very relevant entity in the elderly. It is well known that the prevalence of HF reaches up to 20% among people over 75 years old [1,2]. Most patients with HF are elderly, constituting up to 80% of patients suffering from this disease with both the incidence and prevalence of the condition increasing with age [30]. Age has been associated with a greater risk of cardiovascular events and mortality, especially in patients with HF [31]. We must consider that elderly patients have different clinical profiles than younger ones, characterized by complex comorbidities and frailty. The latter is an independent predictor of adverse outcomes, and it is associated with poorer prognosis in terms of quality of life, hospitalization and mortality [32]. As a matter of fact, we must point out that despite comorbidities and polypharmacy, age does not seem to be associated with lower adherence to medical treatment [33]. In this regard, efforts to maintain strict compliance and improve clinical results are essential.

Although guidelines proclaim that pharmacotherapy and other treatments of HFrEF in elderly patients are recommended to be the same as for younger patients [2,34], in the real world, the use of guideline-directed medical therapy is notoriously lower in older patients despite its potential benefits [2,23,35,36,37]. As an example of this, Euro Heart Failure Survey II reported that the use of ACEi/ARBs in patients over age 80 with HFrEF is approximately 60% at admission due to HF, with this rate rising to 75% at discharge [38]. This percentage is relatively low given that these drugs have been proven to protect against mortality. The use of these drugs is frequently limited by the already mentioned presence of different comorbidities, adverse events and polypharmacy [38]. Elders are also less frequently referred to a cardiologist [39,40]. Altogether, these factors contribute to the lower rate of appropriate HF treatment and may result in lower adherence to current clinical guidelines among elderly patients. In addition, optimal doses are frequently not achieved in these patients despite their positive impact on prognosis [41,42]. Therefore, elderly patients with HF also very often do not benefit from an optimized medical regimen. This underuse is concerning given the growing evidence for each HF-specific drug in this subgroup of patients. Despite the small number of trials that have targeted older HF patients (such as the SENIORS trial [43] or the CIBIS-ELD trial [44]), these trials have reinforced the idea of the clinical benefit and safety of using anti-remodeling drugs and opened the door toward the use of HF-specific drugs in elderly patients with HFrEF. Nevertheless, most of the current data regarding the use of HF drugs in the elderly still come from sub-analyses and observational studies. Some of them have proven to be useful in bringing some light to the use of ACEi/ARBs and BBs in elderly patients, even in the context of frequent clinical situations and comorbidities (such as CKD) [37,45,46,47,48]. Therefore, we still have a real gap in evidence regarding the use of guideline-directed medical therapy in elderly HF patients.

The use of ARNIs is no exception to this situation. Insights from the CHAMP trial show that among patients with HF and > 65 years old, only 12.9% were taking ARNIs [36]. In line with these results, just 14.3% of our patients were taking any dose of sacubitril/valsartan at the end of the follow-up. Moreover, almost 86% of our patients did not have a clear clinical reason for not being on ARNIs. It is quite significant that even with the growing evidence regarding the safe use of ARNIs in this population since 2018, the proportion of patients remains almost the same in different but comparable cohorts [36]. Our study also highlights the reasons for not starting or withdrawing sacubitril/valsartan, with impaired renal function and symptomatic hypotension being the two main ones. Nonetheless, in more than 80% of patients, there was no clear reason for not initiating ARNIs. This could be explained by the lower rate of adherence to clinical guidelines in this subgroup of patients, probably due to the already mentioned factors (polypharmacy, comorbidities, relatively fewer referrals to the cardiologist, etc.).

In our study, we found a significant reduction in cardiovascular events and total mortality associated with the use of ARNIs in our population of very elderly patients with HFrEF. This is even more interesting in the context of low rates of use of sacubitril/valsartan. Another important fact is that our study included a significant population of 616 patients with HFrEF and older than 75 years old. There are few studies directly focused on and with such a cohort size in this range of age [49]. In addition, our cohort comes directly from real-world clinical practice, with a mean age (83 years old) much higher than those from the pivotal trials [10,11,12,21].

Our research presents some limitations. Firstly, it is an observational study, with the subsequent risk of biases. Secondly, it is a single-center study, explaining the relatively small sample size, which may affect the statistical results provided. Another limitation is that we could not ascertain the cause of death in almost 16% of our population because it was not available (we do not have access to the complete history of other centers).

Despite the limitations of our study, it appears unlikely that future randomized trials regarding the use of ARNIs in the elderly will be conducted, especially if we consider the small number of them carried out in the cardiovascular field in the last decade [43,44,49]. Our study addresses clinically relevant endpoints, with a long follow-up period, in a cohort with scarce clinical evidence. As such, the results of this paper are both innovative and potentially applicable to daily clinical practice, regardless of the observational nature of the registry. Further research using real-world data could help to design larger studies and generate more research for this “forgotten” population.

## 5. Conclusions

Based on the findings of our study, treatment with ARNIs in elderly patients presenting with HFrEF was associated with a significant reduction in mortality and cardiovascular events. However, a significant underuse in this population was observed. Those patients not being treated with sacubitril/valsartan did not have a clear contraindication in most of the cases. Our data support that an increased use of ARNIs in this subgroup of patients would probably lead to a significant improvement in prognosis. Nevertheless, more studies focused on this subgroup are needed to confirm these findings.

## Figures and Tables

**Table 1:** Baseline characteristics of the study population.

	Total Population (N = 616)	ARNI (after Propensity Score Matching) (N = 258)		
		Yes (N = 86)	No (N = 172)	*p*-Value
Age, years ± SD	83.3 ± 5.1	80.6 ±3.8	80.9 ± 4.3	NS
Male, n (%)	404 (65.6)	60 (69.8)	115 (66.9)	NS
High blood pressure, n (%)	500 (81.2)	67 (77.9)	139 (80.8)	NS
Diabetes mellitus, n (%)	218 (35.4)	29 (33.7)	62 (36.0)	NS
Hyperlipidemia, n (%)	352 (57.1)	48 (55.8)	93 (54.1)	NS
Current smoker, n (%)	253 (41.1)	41 (47.7)	71 (41.3)	NS
BMI > 30kg/m^2^, n (%)	66 (10.7)	9 (10.5)	28 (16.3)	NS
Chronic lung disease, n (%)	109 (17.7)	16 (18.6)	37 (21.5)	NS
Stroke/TIA, n (%)	100 (16.2)	9 (10.5)	13 (7.6)	NS
Peripheral artery disease, n (%)	78 (12.7)	11 (12.8)	17 (9.9)	NS
Chronic liver disease, n (%)	14 (2.3)	1 (1.2)	3 (1.7)	NS
Chronic kidney disease, n (%)	330 (53.6)	42 (48.8)	74 (43.0)	NS
Cognitive impairment, n (%)	75 (12.1)	0 (0)	11 (8.2)	NS
Functional disability, n (%)	94 (15.3)	8 (9.3)	11 (6.4)	NS
LVEF, % ± SD	28.5 ± 7.8	28.6 ± 7.5	28.4 ± 8.0	NS
Ischemic LV dysfunction, n (%)	287 (53.9)	50 (59.5)	79 (51)	NS
Previous HF admission, n (%)	269 (43.7)	34 (39.5)	78 (45.3)	NS
QRS > 120 ms, n (%)	155 (60.3)	56 (65.9)	99 (57.6)	NS
Sinus rhythm, n (%)	361 (58.7)	50 (41.2)	101 (41.3)	NS
GFR, mL/min/1.73 m^2^, ± SD	56.9 ± 18.4	60.8 ± 14.8	60.4 ± 17.8	NS
Hemodialysis, n (%)	13 (2.1)	0(0)	5 (2.9)	NS
NYHA class				
I–II, n (%)	519 (86.8)	73 (90.1)	149 (87.1)	NS
III–IV, n (%)	79 (13.2)	8 (9.9)	22 (12.9)	NS
Beta-blockers, n (%)	474 (76.9)	78 (90.7)	139 (80.8)	NS
ACEi/ARB, n (%)	342 (55.5)	–	114 (66.3)	–
MRA, n (%)	249 (40.4)	50 (58.1)	78 (45.3)	NS
SGLT2i, n (%)	26 (4.2)	15 (17.4)	7 (4.1)	0.001
Diuretics, n (%)	442 (71.8)	67 (77.7)	123 (71.5)	NS
Digoxin, n (%)	60 (9.7)	4 (4.7)	15 (8.7)	NS
Ivabradine, n (%)	27 (4.4)	7 (8.1)	7 (4.1)	NS
Amiodarone, n (%)	68 (11.0)	10 (11.6)	20 (11.6)	NS
Anticoagulation, n (%)	296 (48.1)	47 (54.7)	88 (51.2)	NS
ICD/CRT, n (%)	65 (16.6)	19 (22.1)	20 (11.6)	NS

ACEi: angiotensin-converting enzyme inhibitor, ARB: angiotensin receptor blocker, BMI: body mass index, CRT: cardiac resynchronization therapy, GFR: glomerular filtration rate, HF: heart failure, ICD: implantable cardioverter defibrillator, LV: left ventricle, LVEF: left ventricular ejection fraction, MRA: mineralocorticoid receptor antagonist, SGLT2i: sodium-glucose cotransporter 2 inhibitors, NS: not significant, NYHA: New York Heart Association, SD: standard deviation, TIA: transient ischemic attack.

**Table 2:** Univariate and multivariate analyses for overall mortality.

	Univariate Analysis		Multivariate Analysis	
	HR	95% CI	HR	95% CI
**Age**	**1.09**	**1.07–1.12**	**1.05**	**1.02–1.08**
Sex	0.97	0.77–1.37		
High blood pressure	1.02	0.76–1.77		
**Diabetes mellitus**	**1.27**	**1.01–1.60**	**1.36**	**1.07–1.72**
Hyperlipidemia	1.10	0.87–1.38		
Chronic lung disease	1.30	0.98–1.71		
Stroke/TIA	1.17	0.87–1.57		
**GFR**	**0.99**	**0.98–0.99**	**0.99**	**0.98–0.99**
**Functional disability**	**0.40**	**0.26–0.61**	**1.28**	**1.04–1.58**
**Ischemic LV dysfunction**	**1.35**	**1.05–1.73**		
**Previous HF admission**	**1.32**	**1.06–1.66**		
QRS > 120 ms	0.84	0.64–1.11		
No sinus rhythm	1.22	0.98–1.54	**1.33**	**1.06–1.68**
**LVEF**	**0.99**	**0.98–1.00**		
**LVEF improvement**	**0.48**	**0.36–0.62**	**0.49**	**0.37–0.64**
NYHA class III–IV	1.26	0.92–1.74		
**Beta-blockers**	**0.53**	**0.41–0.67**	**0.62**	**0.48–0.80**
**ARNI**	**0.27**	**0.16–0.44**	**0.36**	**0.21–0.61**
ACEi/ARB	0.88	0.70–1.10		
MRA	1.09	0.83–1.44		
**SGLT2i**	**0.12**	**0.03–0.50**		
**ICD/CRT**	**0.53**	**0.35–0.82**		

Included variables in the multivariate analysis: age, GFR, diabetes, previous HF, sinus rhythm, QRS duration, functional disability, ACEi/ARBs, beta-blocker, MRA, ARNI, SGLT2i, digoxin, anticoagulation, LVEF improvement and ICD/CRT. ACEi: angiotensin-converting enzyme inhibitor, ARB: angiotensin receptor blocker, CRT: cardiac resynchronization therapy, GFR: glomerular filtration rate, HF: heart failure, ICD: implantable cardioverter defibrillator, LV: left ventricle, LVEF: left ventricular ejection fraction, MRA: mineralocorticoid receptor antagonist, SGLT2i: sodium-glucose cotransporter 2 inhibitors, NYHA: New York Heart Association, TIA: transient ischemic attack. Statistically significant variables are indicated in **bold**.

**Table 3:** Univariate and multivariate analyses for cardiovascular events.

	Univariate Analysis		Multivariate Analysis	
	HR	95% CI	HR	95% CI
**Age**	**1.06**	**1.04–1.08**	**1.04**	**1.02–1.07**
Sex	0.99	0.80–1.22		
High blood pressure	1.16	0.89–1.52		
**Diabetes mellitus**	**1.23**	**1.00–1.51**	**1.32**	**1.07–1.63**
Hyperlipidemia	1.10	0.90–1.35		
Chronic lung disease	1.29	1.00–1.66		
Stroke/TIA	1.15	0.88–1.49		
**GFR**	**0.99**	**0.98–0.99**	**0.99**	**0.98–1.00**
Ischemic LV dysfunction	1.14	0.92–1.42		
**Previous HF admission**	**1.43**	**1.17–1.75**	**1.36**	**1.1–1.68**
QRS > 120 ms	1.09	0.89–1.35		
**Non-sinus rhythm**	**1.28**	**1.05–1.57**	**1.32**	**1.07–1.62**
**LVEF**	**0.99**	**0.98–1.00**		
**LVEF improvement**	**0.67**	**0.53-0.83**	**0.72**	**0.57–0.90**
NYHA class III–IV	1.09	0.81–1.46		
**Beta-blockers**	**0.64**	**0.51–0.79**	**0.70**	**0.49–0.88**
**ARNI**	**0.27**	**0.16–0.44**	**0.69**	**0.49–0.99**
**ACEi/ARB**	**0.72**	**0.59–0.88**	**0.68**	**0.54–0.85**
MRA	1.01	0.82–1.24		
**SGLT2i**	**0.43**	**0.22–0.83**		
ICD/CRT	0.79	0.56–1.11		

Included variables in the multivariate analysis: age, GFR, diabetes, previous HF, sinus rhythm, QRS duration, functional disability, ACEi/ARBs, beta-blocker, MRA, ARNI, SGLT2i, digoxin, anticoagulation, LVEF improvement and ICD/CRT. ACEi: angiotensin-converting enzyme inhibitor, ARB: angiotensin receptor blocker, CRT: cardiac resynchronization therapy, GFR: glomerular filtration rate, HF: heart failure, ICD: implantable cardioverter defibrillator, LV: left ventricle, LVEF: left ventricular ejection fraction, MRA: mineralocorticoid receptor antagonist, SGLT2i: sodium-glucose cotransporter 2 inhibitors, NYHA: New York Heart Association, TIA: transient ischemic attack. Statistically significant variables are indicated in **bold**.

## Data Availability

The data presented in this study are available from the corresponding authors upon reasonable request.
